# The ocean sampling day consortium

**DOI:** 10.1186/s13742-015-0066-5

**Published:** 2015-06-19

**Authors:** Anna Kopf, Mesude Bicak, Renzo Kottmann, Julia Schnetzer, Ivaylo Kostadinov, Katja Lehmann, Antonio Fernandez-Guerra, Christian Jeanthon, Eyal Rahav, Matthias Ullrich, Antje Wichels, Gunnar Gerdts, Paraskevi Polymenakou, Giorgos Kotoulas, Rania Siam, Rehab Z Abdallah, Eva C Sonnenschein, Thierry Cariou, Fergal O’Gara, Stephen Jackson, Sandi Orlic, Michael Steinke, Julia Busch, Bernardo Duarte, Isabel Caçador, João Canning-Clode, Oleksandra Bobrova, Viggo Marteinsson, Eyjolfur Reynisson, Clara Magalhães Loureiro, Gian Marco Luna, Grazia Marina Quero, Carolin R Löscher, Anke Kremp, Marie E DeLorenzo, Lise Øvreås, Jennifer Tolman, Julie LaRoche, Antonella Penna, Marc Frischer, Timothy Davis, Barker Katherine, Christopher P Meyer, Sandra Ramos, Catarina Magalhães, Florence Jude-Lemeilleur, Ma Leopoldina Aguirre-Macedo, Shiao Wang, Nicole Poulton, Scott Jones, Rachel Collin, Jed A Fuhrman, Pascal Conan, Cecilia Alonso, Noga Stambler, Kelly Goodwin, Michael M Yakimov, Federico Baltar, Levente Bodrossy, Jodie Van De Kamp, Dion MF Frampton, Martin Ostrowski, Paul Van Ruth, Paul Malthouse, Simon Claus, Klaas Deneudt, Jonas Mortelmans, Sophie Pitois, David Wallom, Ian Salter, Rodrigo Costa, Declan C Schroeder, Mahrous M Kandil, Valentina Amaral, Florencia Biancalana, Rafael Santana, Maria Luiza Pedrotti, Takashi Yoshida, Hiroyuki Ogata, Tim Ingleton, Kate Munnik, Naiara Rodriguez-Ezpeleta, Veronique Berteaux-Lecellier, Patricia Wecker, Ibon Cancio, Daniel Vaulot, Christina Bienhold, Hassan Ghazal, Bouchra Chaouni, Soumya Essayeh, Sara Ettamimi, El Houcine Zaid, Noureddine Boukhatem, Abderrahim Bouali, Rajaa Chahboune, Said Barrijal, Mohammed Timinouni, Fatima El Otmani, Mohamed Bennani, Marianna Mea, Nadezhda Todorova, Ventzislav Karamfilov, Petra ten Hoopen, Guy Cochrane, Stephane L’Haridon, Kemal Can Bizsel, Alessandro Vezzi, Federico M Lauro, Patrick Martin, Rachelle M Jensen, Jamie Hinks, Susan Gebbels, Riccardo Rosselli, Fabio De Pascale, Riccardo Schiavon, Antonina dos Santos, Emilie Villar, Stéphane Pesant, Bruno Cataletto, Francesca Malfatti, Ranjith Edirisinghe, Jorge A Herrera Silveira, Michele Barbier, Valentina Turk, Tinkara Tinta, Wayne J Fuller, Ilkay Salihoglu, Nedime Serakinci, Mahmut Cerkez Ergoren, Eileen Bresnan, Juan Iriberri, Paul Anders Fronth Nyhus, Edvardsen Bente, Hans Erik Karlsen, Peter N Golyshin, Josep M Gasol, Snejana Moncheva, Nina Dzhembekova, Zackary Johnson, Christopher David Sinigalliano, Maribeth Louise Gidley, Adriana Zingone, Roberto Danovaro, George Tsiamis, Melody S Clark, Ana Cristina Costa, Monia El Bour, Ana M Martins, R Eric Collins, Anne-Lise Ducluzeau, Jonathan Martinez, Mark J Costello, Linda A Amaral-Zettler, Jack A Gilbert, Neil Davies, Dawn Field, Frank Oliver Glöckner

**Affiliations:** 1 Max Planck Institute for Marine Microbiology, Celsiusstrasse 1, D-28359 Bremen, Germany; 2 Jacobs University Bremen gGmbH, Campus Ring 1, D-28759 Bremen, Germany; 3 University of Oxford, 7 Keble Road, OX1 3QG Oxford, Oxfordshire UK; 4 Centre for Ecology & Hydrology, MacLean Building, Benson Lane, Crowmarsh Gifford, OX10 8BB Wallingford, Oxfordshire UK; 5 CNRS & Sorbonne Universités, UPMC Univ Paris 06, Station Biologique, Place Georges Teissier, F-29680 Roscoff, France; 6 Israel Oceanographic and Limnological Research, National Institute of Oceanography, Tel- Shikmona, POB 8030, 31080 Haifa, Israel; 7 Alfred Wegener Institute, Biologische Anstalt Helgoland, Kurpromenade 201, 27498 Helgoland, Germany; 8 Hellenic Centre for Marine Research, Institute of Marine Biology, Biotechnology and Aquaculture, Gournes Pediados, 71500 Heraklion, Crete Greece; 9 Biology Department and YJ-Science and Technology Research Center, American University in Cairo, New Cairo, 11835 Cairo Governorate Egypt; 10 Department of Systems Biology, Technical University of Denmark, Matematiktorvet 301, 2800 Kgs., Lyngby, Denmark; 11 National University of Ireland–University College Cork, Cork, Ireland; 12 Curtin University, Biomedical Sciences, Perth, Western Australia Australia; 13 Aix Marseille Université, CNRS, IGS UMR 7256, 163 Avenue de Luminy, 13288 Marseille, France; 14 Ruđer Bošković Institute, Bijenička cesta 54, 10 000, Zagreb, Croatia; 15 School of Biological Sciences, University of Essex, CO4 3SQ Colchester, Essex UK; 16 Institute for Chemistry and Biology of the Marine Environment (ICBM), Carl von Ossietzky University Oldenburg, Schleusenstrasse 1, 26383 Wilhemshaven, Germany; 17 Marine and Environmental Sciences Centre, Faculty of Sciences of the University of Lisbon, Campo Grande 1749-016, Lisbon, Portugal; 18 Smithsonian Environmental Research Center, 21037 Edgewater, Maryland USA; 19 Department of Microbiology, Virology and Biotechnology, Odessa National II Mechnikov University, Dvoryanskaya str.2, 65082 Odessa, Ukraine; 20 Matis Ltd, Vinlandsleid 12, 113, Reykjavik, Iceland; 21 InBio/CIBIO, Departamento de Biologia da Universidade dos Açores, 9501-801 Ponta Delgada, Portugal; 22 National Research Council, Institute of Marine Sciences (CNR-ISMAR), Castello 2737/f, Arsenale Tesa 104, 30122 Venezia, Italy; 23 Institute of Microbiology/ GEOMAR, Am Botanischen Garten 1-9, 24118 Kiel, Germany; 24 Marine Research Centre, Finnish Environment Institute, Erik Palmenin aukio 1, 00560 Helsinki, Finland; 25 NOAA/National Ocean Service/NCCOS/Center for Coastal Environmental Health & Biomolecular Research Charleston, 29412 South Carolina, USA; 26 Department of Biology, University of Bergen, Thormøhlensgate 53B, 5020 Bergen, Norway; 27 LaRoche Research Group, Department of Biology, Dalhousie University, B3H 4R2 Halifax, Nova Scotia Canada; 28 Department of Biomolecular Sciences, University of Urbino, Viale Trieste 296, 61121 Pesaro, Italy; 29 University of Georgia’s Skidaway Institute of Oceanography, 10 Ocean Science Circle, 31411 Savannah, Georgia USA; 30 NOAA–Great Lakes Environmental Research Laboratory, 4840 S State Road, 48108 Ann Arbor, Michigan USA; 31 National Museum of Natural History, Smithsonian Institution, 10th and Constitution Avenue NW, 20013 Washington, DC USA; 32 CIIMAR, Interdisciplinary Center of Environmental and Marine Research, University of Porto, Rua dos Bragas 289, 4050-123 Porto, Portugal; 33 Station Marine d’Arcachon, CNRS & Univ Bordeaux, 2 rue Professeur Jolyet, F-33120 Arcachon, France; 34 Centro de Investigación y de Estudios Avanzados (CINVESTAV), Unidad Mérida, Carretera Antigua a Progreso Km 6 Cordemex, C.P., 97310 Yucatan, Mexico; 35 Department of Biological Sciences, University of Southern Mississippi, 39406 Hattiesburg, Mississippi USA; 36 Bigelow Laboratory for Ocean Sciences, 60 Bigelow Drive, 04544 East Boothbay, Maine USA; 37 Smithsonian Marine Station, 701 Seaway Drive, 34949 Fort Pierce, Florida USA; 38 Smithsonian Tropical Research Institute, Apartado Postal 0843-03092, Balboa Ancon, Panama; 39 Wrigley Institute for Environmental Studies and Department of Biological Sciences, University of Southern California, 90089-0371 Los Angeles, California USA; 40 Sorbonne Universités, UPMC Univ Paris 06, CNRS, UMR7621, Laboratoire d’Océanographie Microbienne, Observatoire Océanologique, F-66651 Banyuls sur Mer, France; 41 Microbial Ecology of Aquatic Transitional Systems Research Group, Centro Universitario de la Región Este, Universidad de la República, Ruta 15, km 28.500, Rocha, Uruguay; 42 The Mina and Everard Goodman Faculty of Life Sciences, Bar-Ilan University, 5290002 Ramat-Gan, Israel; 43 Interuniversity Institute for Marine Sciences in Eilat, 88103 Eilat, Israel; 44 NOAA Atlantic Oceanographic and Meteorological Laboratory, Ocean Chemistry and Ecosystems Division, 4301 Rickenbacker Causeway, 33149 Miami, Florida USA; 45 Institute for Coastal Marine Environment, IAMC-CNR, Spianata S Raineri, 86 – 98122, Messina, Sicily Italy; 46 Department of Marine Science, University of Otago, PO Box 56, 9054 Dunedin, New Zealand; 47 CSIRO Oceans and Atmosphere Flagship, 7000 Hobart, Tasmania Australia; 48 Department of Chemistry and Biomolecular Science, Macquarie University, 2109 Sydney, Australia; 49 South Australian Research and Development Institute (SARDI) – Aquatic Sciences, PO Box 120, 5022 Henley Beach, South Australia Australia; 50 Flanders Marine Institute, InnovOcean site, Wandelaarkaai 7, 8400 Oostende, Belgium; 51 Centre for Environment, Fisheries and Aquaculture Science (CEFAS), Pakefield Road, NR33 0HT Lowestoft, Suffolk UK; 52 Alfred-Wegener-Institut-Helmholtz-Zentrum für Polar-und Meeresforschung, Am Handelshafen 12, 27570 Bremerhaven, Germany; 53 Microbial Ecology and Evolution Research Group, Centre of Marine Sciences, Algarve University, Gambelas Campus, Building 7, Room 2.77, 8005-139 Faro, Portugal; 54 Marine Biological Association of the UK, Citadel Hill, PL1 2PB Plymouth, Devon UK; 55 Soil and Water Science Department, Faculty of Agriculture, Alexandria University, El-Shatbi, 21545 Alexandria, Egypt; 56 Sorbonne Universités, UPMC Univ Paris 06, CNRS, UMR 7093, LOV, Observatoire océanologique, F-Villefranche-sur-Mer, Paris, France; 57 Marine Biogeochemistry – Argentine Institute of Oceanography, Camino La Carrindanga Km 7,5, 8000 Florida, Bahia Blanca Argentina; 58 Graduate School of Agriculture, Kyoto University, 606-8502 Sakyo-ku, Kyoto Japan; 59 IPMA, Department of Sea and Marine Resources, Avenida de Brasília, s/n, 1449-006 Lisboa, Portugal; 60 Waters, Wetlands and Coasts, New South Wales Office of Environment and Heritage, Sydney South 1232, 59-61 Goulburn Street, 2001 PO Box A290, Sydney, New South Wales Australia; 61 Lwandle Technologies, Black River Park, Fir Road, 7925 Observatory, Cape Town South Africa; 62 Marine Research Division, AZTI, Txatxarramendi ugartea z/g, 48395 Sukarrieta, Bizkaia Spain; 63 CRIOBE, USR3278 CNRS-EPHE-UPVD, LabEx Corail, BP 1013-98729 Papetoai Moorea, French Polynesia; 64 Antarctic and Southern Ocean Studies, University of Tasmania, 7004 Hobart, Tasmania Australia; 65 University of the Basque Country, PO Box 644, E-48080 Bilbao, Basque Country Spain; 66 Gump South Pacific Research Station, University of California Berkeley, BP 244 98728 Moorea, French Polynesia; 67 Polydisciplinary Faculty of Nador, University Mohammed Premier, Selouane, Nador Morocco; 68 Laboratory of Genetics and Biotechnology, University Mohammed Premier, Oujda, Morocco; 69 College of Environmental and Resource Sciences, Zhejiang University, 310058 Hangzhou, China; 70 Polydisciplinary Faculty of Taza, University Sidi Mohammed Ben Abdallah, Fes, Morocco; 71 Faculty of Sciences of Rabat, University Mohammed Fifth Rabat, Rabat, Morocco; 72 Faculté des Sciences et Techniques de Tanger, Université Abdelmalek Essaâdi, Tanger, Morocco; 73 Institute for Genomic and Systems Biology, Bioscience Division, Argonne National Laboratory, 9700 South Cass Avenue, 60439 Argonne, Illinois USA; 74 Pasteur Institute of Morocco, 1 Place Louis Pasteur, 20100 Casablanca, Morocco; 75 Microbiology, Health and Environment Team, Department of Biology, Faculty of Sciences, Chouaib Doukkali University, Rte Ben Maachou, BP 20 Avenue des Facultés, El Jadida, Morocco; 76 University of Chicago, 1101 E 57th Street, 60637 Chicago, Illinois USA; 77 Institute of Biodiversity and Ecosystem Research (IBER), Bulgarian Academy of Sciences, 2 Gagarin Street, 1113 Sofia, Bulgaria; 78 European Molecular Biology Laboratory, European Bioinformatics Institute (EMBL-EBI), Wellcome Trust Genome Campus, Hinxton, CB10 1SD Cambridge, Cambridgeshire UK; 79 Université de Bretagne Occidentale (UBO, UEB), Institut Universitaire Européen de la Mer (IUEM), Place Nicolas Copernic, F-29280 Plouzané, France; 80 Dokuz Eylul University (DEU), Institute of Marine Sciences and Technology (IMST), Baku Bulvard, No: 100, Inciralti, 35340 Izmir, Balcova Turkey; 81 Department of Biology, University of Padova, Via Ugo Bassi 58/B, 35121 Padova, Italy; 82 Singapore Centre for Environmental Life Sciences Engineering, 60 Nanyang Drive, SBS 01N-27, 637551 Singapore, Singapore; 83 Earth Observatory of Singapore, Nanyang Technological University, 50 Nanyang Avenue, 639798 Singapore, Singapore; 84 Indigo V Expeditions, ONE°15 Marina, #01-01, 11 Cove Drive, Sentosa Cove, 098497 Singapore, Singapore; 85 School of Marine Science and Technology, Newcastle University, Dove Marine Laboratory, Cullercoats, NE30 4PZ Tyne and Wear UK; 86 Institute of Marine Science, University of Auckland, Private Bag 92019, Auckland, 1142 New Zealand; 87 PANGAEA - Data Publisher for Earth & Environmental Science, MARUM Center for Marine Environmental Sciences, University Bremen, Hochschulring 18, 28359 Bremen, Germany; 88 OGS, National Institute of Oceanography and Experimental Geophysics, Via Auguste Piccard, 54, 34151, Santa Croce, Trieste, Italy; 89 Department of Physical Sciences, Faculty of Applied Sciences, Rajarata University of Sri Lanka, Mihintale, Sri Lanka; 90 Marine Biological Laboratory, 7 MBL Street, Woods Hole, 02543 Massachusetts, USA; 91 Department of Earth, Environmental, and Planetary Sciences, Brown University, 02912 Providence, Rhode Island USA; 92 Mediterranean Science Commission, 16 Bd de Suisse, 98 000 Monaco, Monaco; 93 Marine Biology Station, National Institute of Biology, Fornače 41, 6330 Piran, Slovenia; 94 Near East University, TRNC Mersin 10, 99138 Nicosia, Cyprus; 95 Department of Oceanography and Fisheries, University of the Azores, PT-9901-862 Horta, Portugal; 96 University of Alaska Fairbanks, Box 757220, 99775 Fairbanks, Alaska USA; 97 University of Hawaii at Manoa, Kewalo Marine Laboratory, 41 Ahui St., Honolulu, 96813 Hawaii, USA; 98 Phytoplankton Ecology, Marine Scotland Marine Laboratory, 375 Victoria Road, AB11 9DB Aberdeen, Aberdeenshire UK; 99 Institut National des Sciences et Technologies de la Mer (INSTM), 28 rue du 2 mars 1934, 2025 Salammbô, Tunisia; 100 Kind of Blue Project ABS and Citizen Science, Gaustadvn 6, 0372 Oslo, Norway; 101 Section for Aquatic Biology and Toxicology, Department of Biosciences, University of Oslo, PO Box 1066, 0316 Blindern, Oslo Norway; 102 Drøbak Field Station, Marine Biology Research station, Biologiveien 2, 1440 Drøbak, Norway; 103 School of Biological Sciences, College of Natural Sciences, Bangor University, LL57 2UW Gwynedd, Bangor UK; 104 Departament de Biologia Marina i Oceanografia, Institut de Ciències del Mar-CSIC, Pg Marítim de la Barceloneta 37-49, E08003 Barcelona, Catalunya Spain; 105 Fridtjof Nansen Institute of Oceanology, First May Street 40, 9000 Varna, Bulgaria; 106 Nicholas School of the Environment and Biology Department, Duke University, 135 Marine Lab Road, 28516 Beaufort, North Carolina USA; 107 Cooperative Institute of Marine and Atmospheric Sciences, Rosenstiel School of Marine & Atmospheric Science, University of Miami, 4600 Rickenbacker Causeway, 33149 Miami, Florida USA; 108 Stazione Zoologica Anton Dohrn, Villa Comunale, 80121 Napoli, Italy; 109 Department of Life and Environmental Sciences, Polytechnic University of Marche, Via Brecce Bianche, 60131 Ancona, Italy; 110 Department of Environmental and Natural Resources Management, University of Patras, 2 Seferi Street, 301 00 Agrinio, Greece; 111 British Antarctic Survey, Natural Environment Research Council, High Cross, Madingley Road, CB3 0ET Cambridge, Cambridgeshire UK

**Keywords:** Ocean sampling day, OSD, Biodiversity, Genomics, Health Index, Bacteria, Microorganism, Metagenomics, Marine, Micro B3, Standards

## Abstract

Ocean Sampling Day was initiated by the EU-funded Micro B3 (Marine Microbial Biodiversity, Bioinformatics, Biotechnology) project to obtain a snapshot of the marine microbial biodiversity and function of the world’s oceans. It is a simultaneous global mega-sequencing campaign aiming to generate the largest standardized microbial data set in a single day. This will be achievable only through the coordinated efforts of an Ocean Sampling Day Consortium, supportive partnerships and networks between sites. This commentary outlines the establishment, function and aims of the Consortium and describes our vision for a sustainable study of marine microbial communities and their embedded functional traits.

## Background

Marine microbes inhabit all marine habitats, are the engines of the ocean’s major biogeochemical cycles, and form the basis of the marine food web [1]. Over the past decades scientists have aimed to understand marine microorganisms, but technical and computational limitations have restricted studies to a local scale. Fortunately, with technological advancements and decreasing sequencing costs, genomic studies have become feasible on a global scale. The first landmark marine metagenome studies were published by the J Craig Venter Institute, beginning with a pilot sampling project in the Sargasso Sea followed by the Global Ocean Sampling (GOS) expedition [2]. The Tara Ocean project expanded this further by integrating the marine genetic, morphological, and functional biodiversity in its environmental context at global ocean scale and at multiple depths [3]. The Micro B3 (Marine Microbial Biodiversity, Bioinformatics, Biotechnology) project now aims to investigate global marine microbial biodiversity and has pioneered the idea to do this on a single orchestrated Ocean Sampling Day (OSD).

## Main text

### Ocean Sampling Day

OSD is a simultaneous, collaborative, global mega-sequencing campaign to analyze marine microbial community composition and functional traits on a single day. On June 21st 2014 – the world’s first major OSD event – scientists around the world collected 155 16S/18S rRNA amplicon data sets, 150 metagenomes, and a rich set of environmental metadata. Standardized procedures, including a centralized hub for laboratory work and data processing via the Micro B3 Information System (Micro B3-IS), assured a high level of consistency and data interoperability [4]. Application of the Marine Microbial Biodiversity, Bioinformatics and Biotechnology (M2B3) standards ensures sustainable data storage and retrieval in respective domain-specific data archives [4]. OSD generated the largest standardized data set on marine microbes taken on a single day, which we consider complementary to other large-scale sequencing projects.

The solstice was chosen to test the hypothesis that diversity negatively correlates with day-length [5]. Data analysis will target three main areas: biodiversity, gene functions, and ecological models. OSD sampling sites are typically located in coastal regions within exclusive economic zones (EEZ). Therefore, the OSD data set provides a unique opportunity to test anthropogenic influences on microbial population ecology. We will perform a multi-level assessment of the human impact on microbial mediated biogeochemical cycles. Questions we would like to answer are: (i) what are the important factors (physical-chemical and biological) in structuring biodiversity patterns and range margins, and (ii) are functions associated with heavy metals, antibiotics or fecal indicators correlated with OSD sites exposed to higher human impact? We are confident that the simultaneous collection of samples will result in the discovery of new ecological patterns providing key information towards understanding environmental vulnerability and resilience.

### Open access strategy and sharing of data

All OSD data are archived and immediately made openly accessible without an embargo period, following the Fort Lauderdale rules for sharing data [6]. Sequence and contextual data are publicly available via the International Nucleotide Sequence Database Collaboration (INSDC) umbrella study PRJEB5129 and at PANGAEA. A model agreement and OSD Data Policy [4] was developed in compliance with the Convention on Biological Diversity and the Nagoya Protocol on Access and Benefit Sharing (ABS) for the utilization on genetic resources in a fair and equitable way. An ABS Helpdesk exists to support OSD participants’ legal questions. Furthermore, the Mediterranean Science Commission (CIESM) developed the CIESM Charter on ABS, which has been endorsed by 391 scientists from 49 countries (as of April 2015).

### The OSD Consortium

At the 16th Genomics Standards Consortium (GSC) meeting in 2014, the OSD community agreed to form the OSD Consortium. Led by the five OSD Coordinators and comprising of up to 130 OSD Site Coordinators and their teams, the OSD Consortium installed the infrastructure and expertise allowing coordinated OSD events to take place. Furthermore, the OSD Consortium aims to foster collaborations and share expertise among and beyond the OSD network, and to connect scientists in a worldwide environmental movement.

### Membership and governance

OSD membership is open to anyone and is earned by participation. Registered participants are provided with privileged access to the OSD network of sites, as well as training activities. OSD samples are prioritized for all types of data generation (as funds and resources allow). In return, participants agree to provide samples according to OSD’s standardized procedures and to work under the umbrella of the OSD Data Policy, which requires open sharing of data and to respect the national legal sampling framework.

### The OSD network of sites

**Fig. 1:**
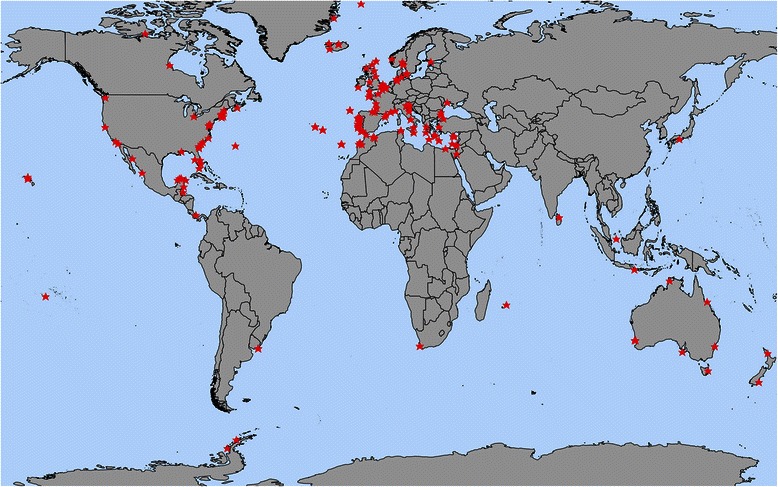
Map of registered sites for OSD 2014

Participants from 191 sampling sites signed up for the main OSD event; these sites range from tropical waters to polar environments (Fig. 1). All major oceanic divisions (Pacific, Atlantic, Indian, Antarctic and Arctic Ocean) and continents are covered with 81 and 37 sites in Europe and North America, respectively. The majority of sites are located in the Northern Hemisphere (172), including 36 sites in the Mediterranean and three sites in the Black Sea.

### OSD partnerships

Endorsement of the community and fruitful partnerships are essential. Supported by the Argonne National Laboratory, the generous cooperation with the Earth Microbiome Project (EMP) [7] enabled us to perform amplicon sequencing for OSD pilot events; these were conducted on each of the solstices in 2012 and 2013. In return, OSD data is EMP compliant and contributes towards construction of a global catalog of microbial diversity [7]. Cooperation with the LifeWatch project secured additional 18S rRNA gene sequencing, while Pacific Bioscience contributed sequencing of near-full-length 16S rRNA gene amplicons and metagenomes from selected OSD sampling sites. Moreover, the partnership with the Smithsonian Institute’s Global Genome Initiative for long-term bioarchiving of all OSD samples enables the community to re-analyze the samples in the future.

### OSD beyond 2014

The OSD Consortium aims to expand in terms of sites and methods, as well as towards multicellular organisms. Future key tasks are to align closely with the Genomic Observatories (GOs) Network [8] towards biocoding the ocean, as well as to secure long-term resources and commitments to create an OSD time-series. The mid-term vision of the OSD Consortium is to generate microbial Essential Biodiversity Variables (EBV) data [9]. The envisioned regular OSD events would qualify for the candidate EBVs “Species populations” and “Community composition” to indicate, for example, vulnerability of ecosystems and climatic impacts on community composition. In the long term such indicators may be incorporated into the Ocean Health Index (OHI) [10], which currently excludes microorganisms from biodiversity assessment due to the lack of reliable data. OSD has the potential to close that gap and amend EBV and OHI by expanding oceanic monitoring towards microbes. This could lead to a global system of harmonized observations to inform scientists and policy-makers.

## Conclusions

This commentary outlines the process for creating, managing and formalizing the OSD Consortium and describes its vision for a sustainable study of marine microbes. As we move forward, we will continue to explore and expand the scope of OSD beyond 2014. The idea of an OSD time-series is still in its early days but incorporating the OSD data set as EBVs and in the OHI is a strong source of motivation since this could pave the way to prioritize scientific research and raise public awareness for the unseen majority of the world’s oceans.

## Abbreviations

ABS: Access and benefit sharing
CIESM: Mediterranean Science Commission (*Commission Internationale pour l'Exploration Scientifique de la Méditerranée*)
EBV: Essential biodiversity variables
EEZ: Exclusive economic zone
EMP: Earth microbiome project
GOs: Genomic observatories
GOS: Global ocean sampling expedition
Micro B3: Marine Microbial Biodiversity, Bioinformatics, Biotechnology
Micro B3-IS: Micro B3 information system
M2B3: Marine microbial biodiversity, bioinformatics and biotechnology data reporting and service standards
EBV: Essential biodiversity variables
OHI: Ocean health index
OSD: Ocean sampling day
rRNA: ribosomal RNA
